# Cultural and Species Differences in Gazing Patterns for Marked and Decorated Objects: A Comparative Eye-Tracking Study

**DOI:** 10.3389/fpsyg.2017.00006

**Published:** 2017-01-23

**Authors:** Cordelia Mühlenbeck, Thomas Jacobsen, Carla Pritsch, Katja Liebal

**Affiliations:** ^1^Department of Education and Psychology, Freie Universität BerlinBerlin, Germany; ^2^Experimental Psychology Unit, Helmut Schmidt University – University of the Federal Armed Forces HamburgHamburg, Germany; ^3^Graduate School “Languages of Emotion”, Freie Universität BerlinBerlin, Germany

**Keywords:** object manipulation, ochre, eye-tracking, external symbolic storage, orangutans

## Abstract

Objects from the Middle Paleolithic period colored with ochre and marked with incisions represent the beginning of non-utilitarian object manipulation in different species of the *Homo* genus. To investigate the visual effects caused by these markings, we compared humans who have different cultural backgrounds (Namibian hunter–gatherers and German city dwellers) to one species of non-human great apes (orangutans) with respect to their perceptions of markings on objects. We used eye-tracking to analyze their fixation patterns and the durations of their fixations on marked and unmarked stones and sticks. In an additional test, humans evaluated the objects regarding their aesthetic preferences. Our hypotheses were that colorful markings help an individual to structure the surrounding world by making certain features of the environment salient, and that aesthetic appreciation should be associated with this structuring. Our results showed that humans fixated on the marked objects longer and used them in the structural processing of the objects and their background, but did not consistently report finding them more beautiful. Orangutans, in contrast, did not distinguish between object and background in their visual processing and did not clearly fixate longer on the markings. Our results suggest that marking behavior is characteristic for humans and evolved as an attention-directing rather than aesthetic benefit.

## Introduction

The emergence of marked objects, either colored or marked with incisions, points to the beginning of human non-utilitarian object manipulation (Bednarik, 1995, 1997) and color symbolism (Roper, 1991; Hovers et al., 2003; Rifkin, 2012; Dayet et al., 2013). Archeological finds documenting this behavior are very old and date back not only to cognitively modern humans, but also to *archaic Homo sapiens, Homo neanderthalensis*, and *Homo erectus* (Rodríguez-Vidal et al., 2014; Joordens et al., 2015). Examples of these findings include non-utilitarian pigment processing with ochre, which is more than 200,000 years old (Mcbrearty and Brooks, 2000), and the use of incisions, with findings about 100,000 years (Hovers et al., 1997; Balter, 2009) to about 75,000 years old in different archeological sites (Henshilwood et al., 2002, 2009). Colored shells about 92,000 years old from Qafzeh Cave in Israel (Hovers et al., 2003; Bar-Yosef Mayer et al., 2009) and shell beads about 82,000 years old from North Africa (Bouzouggar et al., 2007) are also important examples of decorating behavior. Further examples of non-utilitarian manipulated objects from around the same time period include two very old pieces of presumed figurative art that are older than *modern Homo sapiens*: the Venus of Berekhat Ram, with an estimated age of 250,000–280,000 years, and the Venus of Tan-Tan, with an estimated age of 300,000–500,000 years. Investigations of the Venus figurines have not clearly demonstrated that they were shaped by human hands. However, in the case of the Venus of Tan-Tan, researchers have come to the conclusion that its pre-existing strange natural form was used and subsequently changed (Bednarik, 2003), and it is important that the figurine shows traces of ochre, which in any case was applied. The Venus of Berekhat Ram is also assumed to have been developed from a previously existing natural form (d’Errico and Nowell, 2000). All of these examples provide evidence for a gradual development (Mcbrearty and Brooks, 2000) of non-utilitarian object manipulation. The question therefore arises: what were the benefits of this object-marking and shaping behavior? Moreover, how might this behavior be related to the beginning of external symbolic storage? An investigation of visual perceptions of such objects may yield an answer, since the markings are visual attributes. Through markings, different object structures are created and different object–background relations are constructed (Singer and Gray, 1995). The objects emerge from their background and for this reason become special (Dissanayake, 2007).

In connection with neuro-cognitive studies, the term *salience* is especially important, as it has been found that human visual perception is, among other things, salience driven—that is, there is a biased competition between different objects in a visual scene (Desimone and Duncan, 1995). This competition is driven, on the one hand, by the inherent salience of the objects (Yantis, 2005) and, on the other hand, by the influence of top–down attention (Desimone and Duncan, 1995) to focus on the desired perceptual features (for a brief summary see Shinn-Cunningham, 2008). In partial motivation of our study, the question arises whether this salience-driven visual perception is similar among different cultures and different species—a question that has not been investigated to date.

In addition, coloring and decorating behavior and the reception of such coloring and decorating are often accompanied by aesthetic appreciation (Boyd, 2005; Cela-Conde et al., 2009), which in turn is not limited to material items but also involves music, movements, performances, speech, and so on (Dissanayake, 2007). However, although aesthetic appreciation in general is a human universal, the underlying principles that shape aesthetic appreciation are not themselves universal but are influenced by cultural and historic changes (e.g., see Cortissoz, 1913, as an example involving a critical evaluation of van Gogh’s paintings). This leads to the question of whether markings might be bound to aesthetic appreciation *per se*, independent of a specific cultural context and due instead to attention-driving effects. An investigation of this question necessitates combining experimental aesthetics (Fechner, 1897; Martin, 1906; Jacobsen, 2006) with comparative behavioral research. Such a comparative approach has already been used by Morris (1962) and Rensch (1964) to investigate different great ape species’ preferences for patterns and forms, and also in one of our previous studies on the preferences of two different human cultures and orangutans for symmetric and asymmetric patterns (Mühlenbeck et al., 2016).

Our overall hypothesis was that markings, such as those involving ochre, incisions, and decorations, were invented in the Middle Paleolithic period for structuring effects by making the objects more salient and behaviorally relevant for the attention of others (which also corresponds to the findings of Yarbus et al., 1967, that observers repeatedly return to the same important elements of a picture instead of analyzing secondary elements). This resulted in our research hypothesis that the visual perception of marked objects should be different in different species, but not in different human cultures. In addition, we hypothesized that aesthetic appreciation of these markings should be found in humans. To test these hypotheses, we conducted an eye-tracking study with two sorts of stimuli, hand axes, and sticks, which are typical tools of prehistoric humans and great apes. These were always presented in pairs, with an unmarked object next to a marked one. Our purpose was to investigate whether the viewing patterns of the different groups in our study exhibit species- and culture-dependent differences, whether the markings are used in the structural processing of the objects (we will henceforth refer to an individuals’ attentional schematization of the world as ‘structural processing’), and whether the marked objects are fixated upon for a longer time than the unmarked ones. To investigate whether a preference for marked objects is shared by humans independent of their cultural background, we tested adolescents from two different human groups with distinct cultural backgrounds (Namibian hunter-gatherers, Hai//om, or ≠Akhoe Hai//om, and German city dwellers) whose visual exploration behavior should be different based on their visual habituation to either the savannah or industrialized cities. The Hai//om spend most of their daily lives outdoors in the Northern-Namibian Savannah and use allocentric notions—such as north, south, east, and west—to code spatial relations (Haun and Rapold, 2009), while German children spend a considerable amount of time each day inside buildings but are used to navigating in a highly structured, complex urban environment. To test whether the human preference for marked objects is also shared by other species, we compared the performance of the humans with that of a species of non-human great apes, the orangutans—as these are our most distant relatives within the group of great apes. Wild orangutans are highly arboreal and live in the canopy and thus in a densely foliated environment with low visibility (Felton et al., 2003). Although their environment in zoos—which is where our study took place—is very different in regard to the structural complexity and availability of space, they are still familiar with the three-dimensional use of climbing space, and hence, their living environment is very different from those of the human groups. Independent of their different environments, the brains of the human populations differ systematically from those of the orangutans, which should be mentioned here to avoid a misleading impression.

In addition to the eye-tracking test, we conducted an aesthetic preference test in the human groups, in which the participants provided their aesthetic evaluations of a subset of the previously viewed stimuli. The combination of these two tests would give us information about the perceptual benefits that the human development of marking behavior might have had and about whether aesthetic appreciation accompanies the preference for marked objects.

We chose the eye-tracking method because it allows the measurement of visual perception in a non-invasive experimental design where subjects can freely move their head. We measured whether fixation durations, the number of fixations, and the mean duration of single gaze points were influenced by the markings on the objects. Fixation and reading studies (Just and Carpenter, 1980) have revealed that the eye fixates on a stimulus until it has been sufficiently processed and that the longer the fixation duration, the more information is processed (Engbert and Kliegl, 2003). We analyzed the differences in the viewing patterns of the three groups to determine how the marking was used in the visual processing and how the object was perceived in its overall context. Thus, accurate data about attentional allocation to objects could be obtained, as Berlyne urged for psychological aesthetics (Berlyne, 1971, 1974), through use of the eye tracker. We then compared the attention given to an object with the conscious evaluation provided by the participants in the aesthetic preference test.

Some eye-tracking studies have been conducted to compare the viewing behavior of different species (Kobayashi and Kohshima, 2001; Kano et al., 2011; Kano and Call, 2014), including orangutans. Recently, non-invasive eye-tracking techniques have been applied to non-human primates, and great apes in particular, to investigate cognitive processes in a comparison of different species. The studies have focused on the general structure of eye movement (Kano and Tomonaga, 2009; Kano and Tomonaga, 2011a,b; Kano et al., 2011), species-specific social cues (Hattori et al., 2010) and humans’ and apes’ processing of emotional facial expressions (Kano and Tomonaga, 2010). However, while the use of eye-movement recordings is an established paradigm in experimental aesthetics for analyzing different fixation patterns (Buswell, 1935; Brandt, 1945; Locher, 2006) and quantifying differences in the fixation patterns of art-trained and untrained viewers (Nodine et al., 1993), a cross-species comparison using the eye-tracking method is new in this field. In our study, we expected to find differences in the viewing patterns related to the cultural and species differences between the three groups, because their visual exploration of the stimuli should mirror their visual adaptation to different habitats. If our hypothesis was confirmed, the viewing patterns should show that both human groups had developed an ability to treat decorated objects as more important than undecorated ones, and their aesthetic evaluations should be in accordance with their fixation preferences. The highlighting of objects is the basis for the creation of material symbols (as the signifier introduced by De Saussure et al., 2011) and thus for the invention of long-term external symbolic storage. A preference for decorations in the two different cultures, both in the fixation times and in the aesthetic evaluation, could support the idea that visual ordering effects can be accompanied by aesthetic appreciation.

## Materials and Methods

### Participants and Ethics Statement

We tested 27 adolescent members of a Namibian hunter–gatherer group (≠Akhoe Hai//om; mean age 12.3; age range 8–20 years; 9 male, 18 female), 25 German city dwellers from two schools in Hamburg and Berlin, Germany (mean age 13; age range 9–18 years; 12 male, 13 female) and 8 Sumatran orangutans (*Pongo pygmaeus abelii*; mean age 14.5; age range 3–32 years; 3 male, 5 female) at the Wolfgang Köhler Primate Research Centre (WKPRC) of the Max Planck Institute for Evolutionary Anthropology (MPI-EVA) at Leipzig Zoo, Germany.

Our research in Namibia was carried out in strict accordance with the ethical guidelines of the “Working Group of Indigenous Minorities in Southern Africa” (WIMSA) and received their approval. A video clip was used to present the instructions for the study to the participants in their mother tongue and to obtain their informed consent before testing, which was documented on video. It was not possible to obtain written consent because of their lack of reading and writing skills. For minors the informed consent was also obtained by the parents of the children. The participants were all recruited by and tested in a room of the school at their village. We used opportunity sampling – so, whoever was willing to participate was tested, which explains the large variation in age. This was not possible otherwise, due to the migration behavior of the families.

Research at the German schools was conducted in accordance with the ethical recommendations of the Deutsche Gesellschaft für Psychologie (DGPs; German Psychological Association), and the ethical guidelines of the research institution (Freie Universität Berlin). After the pupils agreed to participate, their parents gave their written informed consent to the participation in this study. The pupils were tested in rooms located at their schools.

Hence, informed consent was obtained for all human subjects in accordance with the Declaration of Helsinki.

Research at the WKPRC was conducted in accordance with the recommendations of the Weatherall report “The Use of Non-human Primates in Research” (Weatherall, 2006). The WKPRC orangutans were housed in semi-natural indoor (230 m × 230 m) and outdoor (1680 m × 1680 m) enclosures with regular feedings, daily enrichment and water ad lib. All orangutans were from the same group and voluntarily participated in the study, were able to stop participating at any time and were never food- or water-deprived. The research was conducted in observation rooms (25 m × 25 m). At the WKPRC, no medical, toxicological or neurobiological research of any kind is conducted. The research was non-invasive and strictly adhered to the legal requirements of Germany. The study was ethically approved by an internal committee at the Max Planck Institute for Evolutionary Anthropology (members of the committee are Prof. M. Tomasello, Dr. J. Call, Dr. D. Hanus, veterinarian Dr. A. Bernhard, head keeper F. Schellhardt and assistant head keeper M. Lohse). The study was also carried out in strict accordance with the “EAZA Minimum Standards for the Accommodation and Care of Animals in Zoos and Aquaria” (EAZA, 2008/2014), the “WAZA Ethical Guidelines for the Conduct of Research on Animals by Zoos and Aquariums” (WAZA, 2005) and the “Guidelines for the Treatment of Animals in Behavioral Research and Teaching” of the Association for the Study of Animal Behavior (ASAB, 2006).

### Apparatus

**FIGURE 1:**
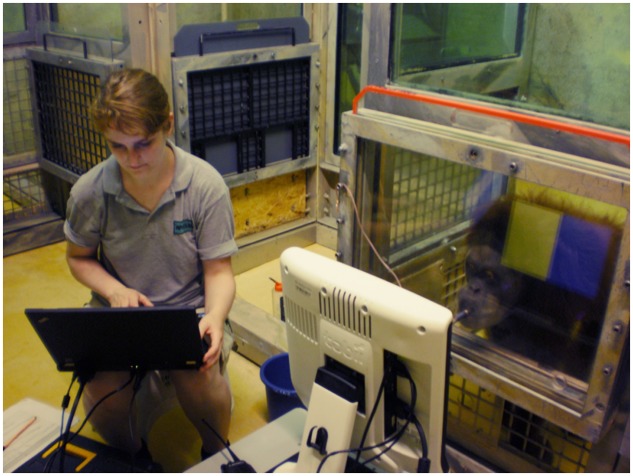
**Testing room in the WKPRC**. An adult orangutan is looking at the eye tracker while drinking juice through a flexible tube attached to the plexiglass panel. The experimenter is operating the eye tracker via the laptop. Image credit: Cordelia Mühlenbeck.

A screen-based Tobii T 60 eye tracker (60 Hz, Tobii Technology) integrated in a 17-in TFT monitor (screen resolution 1280 × 1024 pixels) with Tobii software (version 2.1) was used to test the German group and the orangutans (see **Figure 1**). Because the Namibian group lived in a remote area, which required more mobile equipment, a Tobii X2 60 with the same Tobii software was used in combination with a laptop (screen size 15.6′, screen resolution 1366 × 768 pixels, but same stimuli size as on T 60 monitor). The stimuli were presented on the monitor (Tobii T 60) or the laptop screen (Tobii X2 60), both operated by the experimenter from a laptop next to the monitor/laptop screen (see **Figure 1**). With this non-invasive technique, the participants were able to move their heads freely, which allowed for natural behavior and thereby increased the ecological validity. The programming of the eye tracker allowed for head movements and could re-catch the eyes after a loss of contact, continuing with the recording.

The orangutans sat in a testing room separated from the experimenter by a plexiglass panel (**Figure 1**), allowing the eye tracker to capture the eye movements through the panel. The distance between the eye tracker and the participant was adjusted to 60–70 cm before each trial, the eye tracker’s most accurate recording distance, for capturing the participants’ eye movements. To position the apes in front of the panel and to capture their attention, the experimenter offered them juice through a small hole in the plexiglass panel.

### Stimuli

**FIGURE 2:**
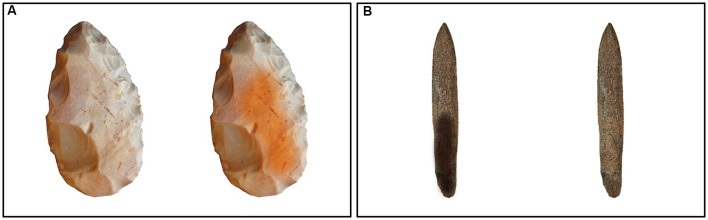
**Example of a hand axe stimulus (A)** and a stick stimulus **(B)**. Unmarked objects were always combined with marked copies of themselves (hand axes: marked on right, sticks: marked on left). The position of marked objects was randomized. Image Credits: J. R. Katzman, Aggbach’s Paleolithic Blog, hand axes: (Katzman, 2012b) and sticks: (Katzman, 2012a).

Our goal was to get matched objects with and without markings and incisions. Images from archeological databases served as templates. We digitally removed marks in some cases and added them in others. These systematically varied in material, complexity and form. The markings and incisions were also added to the objects in various positions to represent different structures and not to predetermine a single structure, because the purpose of this study was to find out whether *any* markings are used in the visual processing of the stimuli. The total set of stimuli comprised 60 hand axes and 60 sticks. In each stimulus the objects were combined with a marked or unmarked copy of itself and not with other objects (see **Figure 2**). The markings on the objects were created with an image-editing program (GIMP version 2.8). To better balance the cultural knowledge, some markings were produced in accordance with ornaments of southwest African hunter–gatherer populations (Sütterlin, 2003). The image-editing program was used to position all of the objects in the center of the image with the background set to transparent, to avoid any position or background color interference. The size of all stimuli was 880 × 547 pixels. For the human participants, the two conditions were presented successively (and randomized), and the stimuli of each condition were presented in different randomizations. For the apes, the 60 stimuli of each condition were divided into four subsets of 15 stimuli and then the subsets were presented in different randomizations. The position of the marked object on the left or on the right side was also balanced and randomized in the sequence to avoid any effect of reading direction or habituation.

For the aesthetic preference test, the 60 stimuli of both the hand axes and the sticks were divided into 4 subsets of 15 stimuli. The participants were assigned to these blocks in 4 equally large subgroups. The presentation of only one subset was due to reasons of time. The participants were asked which of the two objects—marked or unmarked—they found more beautiful and to point to that object. After they pointed, the next pair of objects was presented. Their pointing gestures were documented on video for further analysis.

### Calibration and Testing Procedure

Because spontaneous gazing behavior was recorded, training was not necessary. All participants had some experience with watching computer screens; both the great apes and the Namibians had previously participated in eye-tracker studies, while the Germans were generally more familiar with looking at (TV) screens. Before each testing trial, the participants were verbally instructed to watch the pictures naturally, and the participants’ gazes were then calibrated. A manually changed two-point calibration was conducted for the orangutans and an automated five-point calibration was conducted for the humans, to adjust the eye tracker to their eyes to increase tracking accuracy. Recalibration was conducted until the calibration showed almost the same accuracy for all participants. Only then did the test trial begin. For all participants, each stimulus was presented for 3 s, followed by a fixation cross presented for 0.5 s to recenter the participants’ eyes in the middle of the picture before the next stimulus was presented. For humans, the 60 stimuli were presented in a single test for each condition, with each test followed by the aesthetic preference test, for a total period of 4.5–5 min for each condition, depending on how much time the participants needed to finish the aesthetic preference task. Humans received both conditions on a single day (in randomized order), while the orangutans were presented with only one of the four subtests of the 60 stimuli per day because of their shorter attention span. During the testing, the apes received food reward to hold their attention. Each session for the apes lasted between 10 and 15 min, including the calibration. Because orangutans sometimes ignored trials completely (due to their shorter attention span), their measurements had to be repeated to ensure that the number of stimuli observed by them was similar to that of the human groups (see below under data analysis for further description). In the human groups, no measurements were repeated.

### Data Analysis

The eye-tracking cameras detected the distance of the eyes and their angular position with a frequency of 60 Hz and matched them to the coordinate system of the stimuli on the monitor. The angular position was used for calculating the gaze points, which then were aggregated to fixations when they cumulated for duration of 100 ms, based on a radius of 50 pixels. No corrections of the raw tracking data were conducted. For the aesthetic preference test, 9 of the 27 Namibian participants had to be excluded from the data analysis because they did not understand the task or because of software malfunctions (1 participant). Due to the repeated measurements in the orangutan group, the first recording of each stimulus which displayed a viewing pattern of a minimum of two gaze points was used for analysis. This guaranteed that for the orangutan participants, only the process of viewing unknown objects was analyzed, in order to be comparable to the viewing of the human participants, who had seen each stimulus only once. Later recordings for the orangutans were not used for the analysis because they represented visual processing of an already known stimulus.

For the statistical analysis of participants’ viewing behavior, we considered large areas of interest (AOI), splitting the screen down the middle and counting fixations on the side of the marked object versus that of the unmarked object. For the spatial analysis of the viewing behavior, we compared the data for this large AOI to those for a smaller AOI which was drawn closely around the object, 20 pixels larger than the object’s border. The smaller AOI was used for the spatial analysis because the difference between the small and large AOIs gives us information about the gaze points lying outside the object and thus information about the radius in which the objects were scanned by the three groups. For the temporal analysis of the fixation preference, we used the large AOI and included the fixation points around the objects in the analysis because they represent the processing of the object–background relation. Data tables can be found in the Supplementary Material section.

### Statistical Methods

To investigate our hypotheses we analyzed the viewing behavior of the three groups for three main aspects. First, we analyzed the temporal processing of the stimuli in regard to the fixation preference (results see Fixation Preference Value), the number of fixation points (results see Number of Fixations) and the mean duration of the single gaze points (results see Mean Fixation Duration of Gaze Points). Second, we analyzed the matching of the fixation preference with the aesthetic preference in the two human groups (results see Aesthetic Preference Test), and, third, we analyzed the spatial aspects of their viewing behavior to find out how the marked and unmarked objects were processed in combination with their background (results see Spatial Analysis).

As a first analysis, descriptive statistics (mean values) of the eye movement data were computed. For further analyses we chose to use multilevel-models (general linear mixed models – glmm) in accordance with the hierarchical structure of our data. Our data structure was hierarchical in different respects: first, we had the hierarchy of individuals and groups; in addition, for questions two and three there were also several data points (several fixation points) for each trial (60 pictures). With multi-level analysis it is possible to take the influences of the different trials and the influences of the different individuals into account (Barr, 2008), because the random effects of both can be included into the analysis. This leads to relative rather than absolute values.

To test whether the fixation duration for the hand axes and sticks was influenced by the markings on the objects and the cultural and species differences between the three groups, we first generated a *fixation preference value*, which represents the proportion of the fixation duration directed toward the marked object in regard to the total fixation duration on the stimulus, with a value above zero representing a preference for the marked objects and below zero for the unmarked objects. It was calculated as follows: fixation time on marked object divided by total fixation time on stimulus, minus 0.5. By subtracting 0.5 the values for the preference for marked objects are set above zero and for unmarked objects below zero, for better visibility. To test the significance of this fixation preference value, we used a general linear mixed model (e.g., Baayen, 2008; Bolker et al., 2009) which, in the null model, comprised only the fixation preference value as the dependent variable with the fixations of all subjects in the intercept and the factor subject as random effects [model 1 (a) and (b); for a definition of the random intercept model see Snijders and Bosker, 1999]. In the full model we included group (Namibians, Germans, and orangutans) and gender as fixed effects and subject as random effects. The age of the participants was excluded from the analysis due to theoretical reasons, because the human groups fell into one age range which was not comparable with the different age range of the orangutans. We inspected the data for a possible side bias of the participants, which we did not find. Correlations between the fixed effects were not assumed. We checked whether the assumptions of normally distributed and homogeneous residuals were fulfilled by visually inspecting a *qq*-plot and the residuals plotted against fitted values; both of these indicated no obvious deviations from these assumptions. We examined the model stability (function ‘influence’ of the R-package influence.ME, Nieuwenhuis et al., 2012) by inspecting dfbetas, cook’s distance and the sigtest for both the hand axes and the sticks, and these revealed that no influential cases existed. Variance inflation factors (VIFs; e.g., Field, 2013) were derived by applying the function ‘vif’ of the R-package car (Fox and Weisberg, 2011) to a standard linear model excluding the random effects, and these indicated that collinearity was not an issue (i.e., that the predictors were not correlated).

To test whether the number of fixations [model 2 (a) and (b)] and the mean fixation duration of the single gaze points [model 3 (a) and (b)] on the hand axes and sticks were influenced by the markings on the objects and the differences between the three groups, we again used two general linear mixed models. In both of these, we included into the full models objects (marked or unmarked), group and gender as fixed effects with a cross-level interaction (Baayen et al., 2008) between objects and group (to obtain the differences in the respective influences), and stimulus number and subject as cross-classified levels (cross-classified-models, Hox, 2002) with random slopes of objects in both. First, we ran a reduced-full-model comparison, where we excluded our main fixed effects objects, group and the cross-level-interaction between objects and group to test which model was significantly better. To identify the specific influence of each factor, we ran again a reduced-full-model comparison with R function *drop1* (argument ‘test’ set to ‘Chisq’), which excludes each fixed effect after another. Since there was a distribution asymmetry in the data of the mean duration of the single gaze points (for both hand axes and sticks), which was similar in all three groups, we log-transformed the dependent variable (mean fixation duration) for model 3 (a) and (b) to achieve a more symmetrical distribution and hence better interpretability. Correlations between the fixed effects were not assumed. Again, no obvious deviations from the model assumptions were found (we examined *qq*-plot and residuals against fitted values for normally distributed and homogeneous residuals). The test for model stability (using the R-package influence.ME with function ‘influence,’ Nieuwenhuis et al., 2012), inspecting dfbetas, cook’s distance and sigtest for both the hand axes and the sticks revealed that according to classical cut-off criteria some participants had an influence, but according to content-based criteria they were classified as not excludable. In models 2 (a) and (b) and 3 (a) and (b), we also analyzed the VIFs to test whether collinearity existed, and they indicated that this was not the case. All models were fitted in R (R Core Team, 2013) using the function ‘lmer’ of the R-package lme4 (Bates et al., 2013). To achieve more reliable *p*-values in the full–reduced model comparison, the model was fitted using maximum likelihood (rather than restricted maximum likelihood, Bolker et al., 2009), and its significance was established using a likelihood ratio test (the R function ‘anova’ with the argument ‘test’ set to ‘Chisq’). The effect size of the variables was based on likelihood ratio tests comparing the full with the respective reduced models (e.g., Barr et al., 2013).

### Reliability

To ensure reliability for the analysis of the video data with the pointing gestures of the aesthetic preference test, a person unfamiliar with the purpose of this study coded 20% of the data. Cohen’s kappa was used to measure the degree of concordance. All measured kappa were between 0.93 and 1, which corresponds to an almost perfect level of agreement (Landis and Koch, 1977).

## Results

### Fixation Preference Value

The full–null model comparison revealed that for the fixation preference for the hand axes [model 1 (a), **Table 1**], the full model was significantly better than the null model (likelihood ratio test: χ^2^ = 7.91, *df* = 3, *p* = 0.048), which indicates an effect of group or gender on the fixation preference. To determine which of these factors had an effect we compared the full with the respective reduced models based on likelihood ratio tests (group: χ^2^ = 7.75, *df* = 2, *p* = 0.021). Gender had no effect (gender: χ^2^ = 0.00, *df* = 1, *p* = 0.983). Since gender had no effect, we excluded it from our further analysis. Still, it has to be noted that regarding our dependent variable fixation preference value, the null model already gave us information about our main hypothesis, revealing a general preference for marked hand axes in all three groups (estimate: 0.05; *SE*: 0.01; *t*-value: 5.32; *p* < 0.001), because it represents the proportion of the fixation duration directed toward the marked object in regard to the total fixation duration on the stimulus, as described in the statistical methods section. A value above zero represents a preference for marked objects and below zero for unmarked objects.

**Table 1:** Hand axes and sticks: general linear mixed models for fixation preference value

	Germans	Namibians	Orangutans
**Model 1 (a) Fixation preference Hand axes**			
	Intercept		
Estimate	0.08	-0.05	-0.03
CI lower	0.05	-0.09	-0.09
CI upper	0.10	-0.02	0.02
*SE*	0.01	0.02	0.03
*t*-value	5.90	-2.90	-1.18
**Model 1 (b) Fixation preference Sticks**			
	Intercept		
Estimate	0.12	-0.06	-0.07
CI lower	0.10	-0.10	-0.13
CI upper	0.15	-0.02	-0.01
*SE*	0.01	0.02	0.03
*t*-value	8.99	-3.26	-2.33

*The table shows the results for the two models (hand axes and sticks) for the fixation preference value, comprising the fixation preference value as dependent variable, group (Namibians, Germans, and orangutans) as fixed effects and subject as random effects. Gender was excluded from the analysis, after it was found out to have no effect*.

**FIGURE 3:**
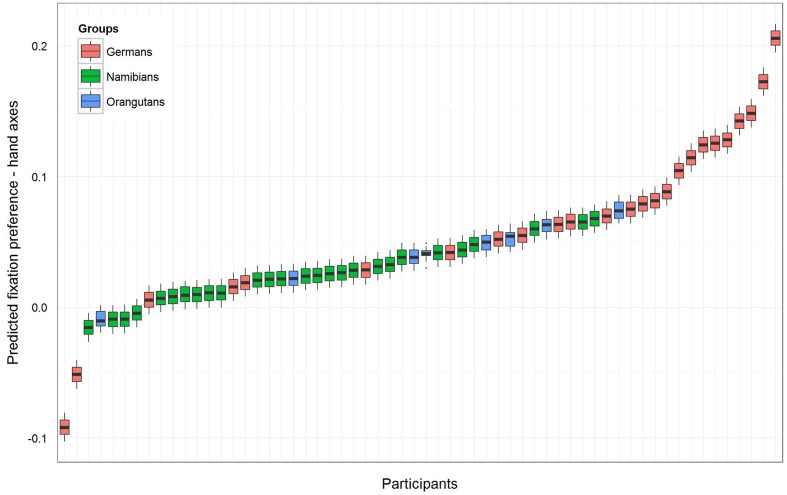
**Hand axes-predicted fixation preference value for each participant**. The figure displays the model for the fixation preference value. A value above zero represents a longer fixation on the marked hand axes, and a value under zero on the unmarked objects. The fixation preference for marked objects was the strongest in the German group, where all participants except two had a value above zero. All but four of the Namibian participants showed a fixation preference value above zero, but the values are lower than those of the Germans and were only almost significant (see **Table 1**). All of the orangutans but one showed fixation preferences above zero, but this revealed no significance (**Table 1**).

To receive the fixation preference value for each level of group we releveled the reference level of the factor group in the intercept to get informative *t*-values and their corresponding *p*-values. Since gender had no effect and was excluded from the further analysis, we calculated the *t*-values and *p*-values for a model comprising only group and the random effects (Namibians: estimate: 0.02, *SE*: 0.01, *t*-value: 1.94, *p* = 0.057; Germans: estimate: 0.08, *SE*: 0.01, *t*-value: 5.90, *p* < 0.001; orangutans: estimate: 0.04, *SE*: 0.03, *t*-value: 1.64, *p* = 0.107). This shows that the fixation preference value of the orangutans is not significant, for the Namibians it is almost significant and for the Germans it is highly significant. **Figure 3** shows the predicted fixation preference value (calculated with the model) broken down for each participant for the hand axes.

**FIGURE 4:**
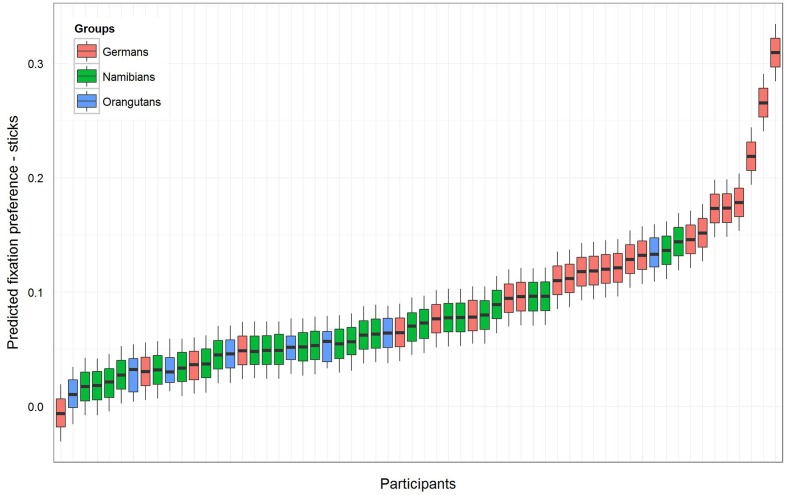
**Sticks-predicted fixation preference value for each participant**. Same representation as in **Figure 3** with an even greater effect for marked sticks. All participants except one German have a value above zero, but in the German group the fixation preference range from zero (equal preference) to 0.5 (preference only for marked objects) is the widest. In both human groups the fixation preference was significant, and in the orangutan group it was only almost significant (see **Table 1**).

For the sticks, the same procedure was maintained as described above. The null model [model 1 (b), **Table 1**] revealed an even higher general preference for the marked objects (estimate: 0.09; *SE*: 0.01; *t*-value: 8.72; *p* = 0). The full–null model comparison revealed that the full model was significant (likelihood ratio test: χ^2^ = 11.36, *df* = 3, *p* = 0.010). A comparison of the full with the respective reduced models based on likelihood ratio tests (Barr et al., 2013) revealed that group had a clear and even stronger effect than was found for the hand axes (χ^2^ = 11.34, *df* = 2, *p* = 0.003) and that gender had no effect (χ^2^ = 0.08, *df* = 1, *p* = 0.784). The respective *t*-values and *p*-values for each level of group (using the same procedure as described above for the hand axes) revealed that the marking effect already found for the hand axes was even stronger here, this time also for the orangutans. The *t*-values and *p*-values were again calculated with a model excluding gender (Namibians: estimate: 0.06, *SE*: 0.01, *t*-value: 4.63, *p* < 0.001; Germans: estimate: 0.12, *SE*: 0.01, *t*-value: 8.99, *p* < 0.001; orangutans: estimate: 0.05, *SE*: 0.03, *t*-value: 1.95, *p* = 0.056). **Figure 4** shows the predicted fixation preference value for each participant for the sticks.

### Number of Fixations

**FIGURE 5:**
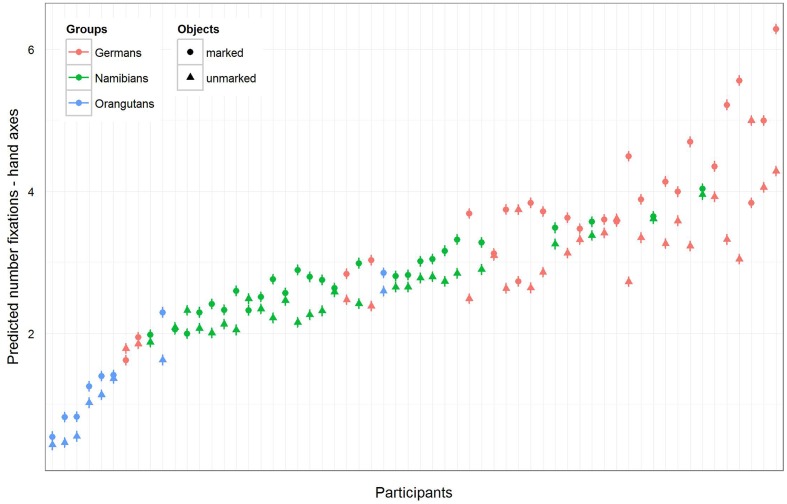
**Hand axes-predicted number of fixations for each participant**. The figure displays the model for the number of fixations for the three groups (highlighted by the different colors) and for each single participant in accordance with the multi-level structure of our analysis. The general number of fixations was the greatest in the German group and the least in the orangutan group, but almost all participants in the three groups showed a higher number of fixations on marked hand axes.

The full–reduced model comparison for the number of fixations [model 2 (a), **Table 2**, with the reduced model comprising only gender and the random effects] revealed that the full model for the hand axes was highly significant (χ^2^ = 428.7, *df* = 9, *p* < 0.001), which indicates an effect for objects, group or the cross-level-interaction between objects and group. With the identification of the influencing factors (R function *drop1*) we confirmed this effect for the cross-level-interaction between objects and group (objects–group interaction: χ^2^ = 7.10, *df* = 2, *p* = 0.029) and found no effect for gender (χ^2^ = 1.68, *df* = 1, *p* = 0.194). Hence, gender was excluded from further analysis. The estimates and their confidence intervals in the group–objects interaction show that for all groups there was a slight effect in the number of gaze points for the marked objects, with the strongest effect in the German group (**Table 2**, **Figure 5**). This means that within the three groups (depicted in **Figure 5** by the different colors), most of the participants tended to have more fixations on the marked objects, with the strongest difference between the numbers of fixations for marked and for unmarked hand axes in the German group. In general, the greatest number of fixations was found in the German group and the smallest in the orangutan group. **Figure 5** provides information regarding the differences in the three groups (different colors), but also in the single subjects (single data points) to illustrate the different levels of analyses we described in the data analysis section. **Table 2** presents the results for the models [hand axes and sticks – models 2 (a) and (b)] without gender, because it was excluded after identified as having no effect.

**Table 2:** Hand axes and sticks: general linear mixed models for number of fixations.

	Germans		Namibians		Orangutans	
Objects	Marked	Unmarked	Marked	Unmarked	Marked	Unmarked
**Model 2 (a) Hand axes Number fixations**
	Intercept				
Estimate	3.86	-0.72	-1.06	0.50	-2.45	0.47
CI lower	3.52	-1.01	-1.52	0.12	-3.13	-0.09
CI upper	4.21	-0.43	-0.60	0.88	-1.78	1.03
*SE*	0.17	0.15	0.23	0.19	0.34	0.28
*t*-value	22.50	-4.89	-4.58	2.63	-7.23	1.68
**Model 2 (b) Sticks Number fixations**
	Intercept				
Estimate	4.09	-1.19	-1.09	0.67	-2.72	1.01
CI lower	3.75	-1.45	-1.56	0.34	-3.39	0.53
CI upper	4.43	-0.92	-0.63	0.99	-2.04	1.48
*SE*	0.17	0.13	0.23	0.16	0.34	0.24
*t*-value	23.75	-8.92	-4.69	4.11	-7.98	4.23

*The table shows the results for the two models (hand axes and sticks) for the number of fixations. They comprised number of fixations as dependent variable, objects (marked or unmarked) and group as fixed effects with a cross-level interaction between objects and group (to obtain the differences in the respective influences), and stimulus number and subject as cross-classified levels with random slopes of objects in both. Gender was excluded from these models after identified as having no effect*.

**FIGURE 6:**
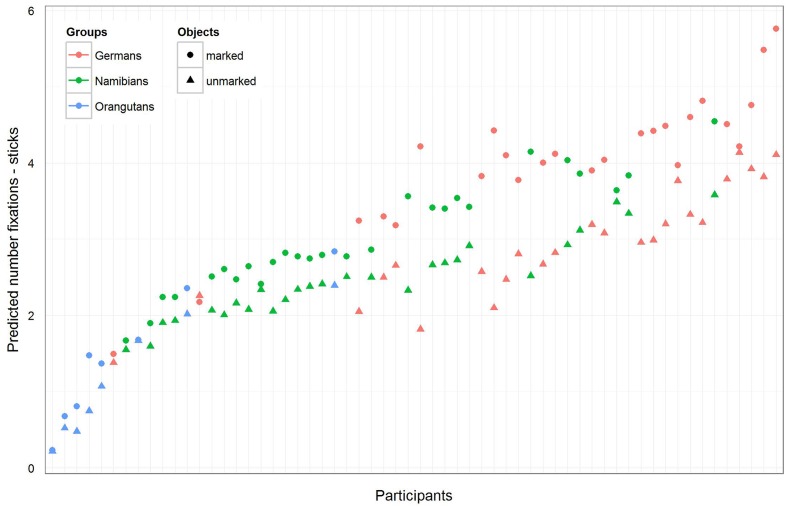
**Sticks-predicted number of fixations for each participant**. Same representation as in **Figure 5**. Again, the general number of fixations was the greatest in the German group, and all participants in the three groups showed a greater number of fixations on the marked sticks. Please note that also here error bars were calculated, but are too small to be visible.

The full–reduced model comparison of the number of fixations for the sticks [model 2 (b)] revealed that the full model was highly significant (χ^2^ = 657.82, *df* = 9, *p* < 0.001), which, again, indicates an effect for objects, group or the cross-level-interaction between objects and group. With the identification of the influencing factors (R function *drop1*) we confirmed the effect for the cross-level interaction (objects–group interaction: χ^2^ = 21.37, *df* = 2, *p* < 0.001) and revealed no effect for gender (χ^2^ = 0.52, *df* = 1, *p* = 0.471). Hence, gender was again excluded from the further analysis. The estimated values and confidence intervals (**Table 2**) of the group–objects interaction show here as well that within the three groups, almost all participants had more fixations on the marked sticks than on the unmarked ones. These results can also be seen in **Figure 6**, which shows that the difference between the numbers of fixations for marked sticks and for unmarked sticks is again the greatest in the German group and is even clearer than already found for the hand axes. Also for the sticks, the Germans had the greatest number of fixations and the orangutans the smallest.

### Mean Fixation Duration of Gaze Points

**FIGURE 7:**
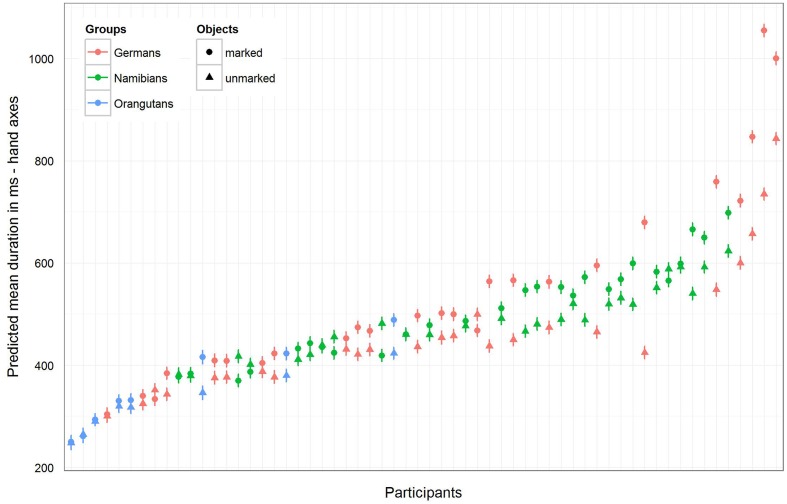
**Hand axes-predicted mean duration of gaze points for each participant**. The figure displays the model for the mean duration of the gaze points with non-log-transformed dependent variable to show the mean duration (in ms) of the single gaze points for the three groups (highlighted by the different colors) and for the single participants. In general, the mean gaze point durations were the longest in the German group and the shortest in the orangutan group. Almost all participants in the three groups showed a longer gaze point duration for the marked hand axes.

For the hand axes, the full–reduced model comparison of the mean fixation duration for the single gaze points [model 3 (a), **Table 3**; again, only gender and the random effects were included in the reduced model] revealed that the full model was highly significant (χ^2^ = 142.51, *df* = 9, *p* < 0.001), which, also here, indicates an effect for objects, group or the cross-level-interaction between objects and group. The identification of the influencing factors confirmed the effect for the group–objects interaction and revealed, also here, no effect for gender (objects–group interaction: χ^2^ = 18.15, *df* = 2, *p* = 0.000; gender: χ^2^ = 0.32; *df* = 1; *p* = 0.575). Gender was, also here, excluded from the further analysis. The estimates for the three groups show that the two human groups had a similar mean gaze point duration which was greater than that of the orangutans (**Table 3**). The estimates and their confidence intervals in the group–objects interaction show that the mean duration of the single gaze points was longer for marked objects than for unmarked objects for most participants of the three groups, with the clearest difference in the German group. **Figure 7** illustrates the differences in the three groups (highlighted by the different colors) and in the single participants regarding the mean duration of the single gaze points on marked and unmarked hand axes. **Table 3** [model 3 (a)] presents the results for the hand axes again without gender, because it was excluded after identified as having no effect. The results for the mean gaze points were calculated with a log-transformed dependent variable as described in the Section “Materials and Methods.”

**Table 3:** Hand axes and sticks: general linear mixed models for mean fixation duration of the single gaze points.

	Germans		Namibians		Orangutans	
**Objects**	**Marked**	**Unmarked**	**Marked**	**Unmarked**	**Marked**	**Unmarked**

**Model 3 (a) Hand axes Mean fixation duration gaze points**
	Intercept				
Estimate	6.10	-0.16	-0.01	0.15	-0.50	0.23
CI lower	6.01	-0.21	-0.13	0.07	-0.69	0.10
CI upper	6.19	-0.10	0.11	0.22	-0.31	0.37
*SE*	0.05	0.03	0.06	0.04	0.09	0.07
*t*-value	135.24	-5.29	-0.14	3.77	-5.31	3.50
**Model 3 (b) Sticks Mean fixation duration gaze points**
	Intercept				
Estimate	6.10	-0.21	0.06	0.10	-0.49	0.06
CI lower	6.02	-0.29	-0.06	-0.00	-0.67	-0.10
CI upper	6.19	-0.14	0.17	0.19	-0.31	0.22
*SE*	0.04	0.04	0.06	0.05	0.09	0.08
*t*-value	138.16	-5.60	0.94	1.93	-5.40	0.73

*The table shows the results for the two models (hand axes and sticks) for the mean fixation duration of the single gaze points. They comprised mean fixation duration as dependent variable, objects (marked or unmarked) and group as fixed effects with a cross-level interaction between objects and group (to obtain the differences in the respective influences), and stimulus number and subject as cross-classified levels with random slopes of objects in both. Gender was excluded from these models after identified as having no effect*.

**FIGURE 8:**
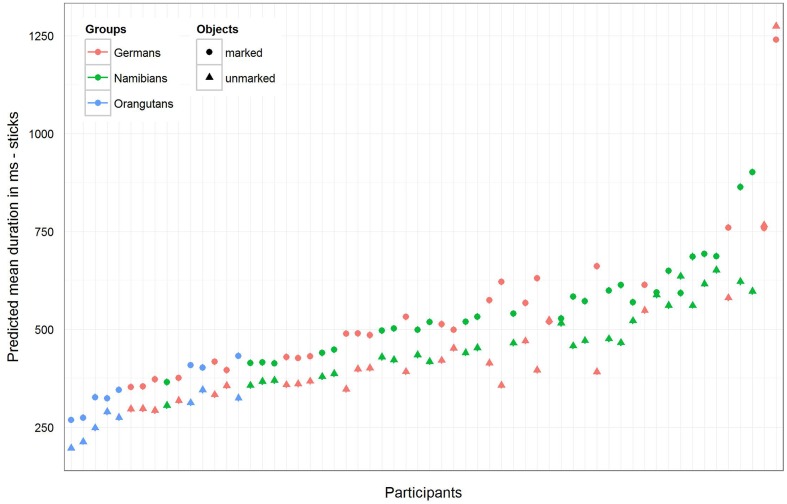
**Sticks-predicted mean duration of gaze points for each participant**. Same representation as in **Figure 7** (non-log-transformed dependent variable). Here the general mean gaze point duration was similar in the German and Namibian groups. Almost all participants in the three groups showed longer mean gaze points for the marked sticks.

For the sticks, the full–reduced model comparison of the mean gaze point duration revealed that the full model was highly significant (χ^2^ = 351.28, *df* = 9, *p* < 0.001), which, again, indicates an effect for objects, group or the cross-level-interaction between objects and group. But, here the identification of the influencing factors could not confirm this effect for the group–objects interaction, which means that the effect must lie in the fixed effects objects and/or group. It revealed, also, that there was no effect for gender (objects–group interaction: χ^2^ = 3.6, *df* = 2, *p* = 0.166; gender: χ^2^ = 0.27, *df* = 1, *p* = 0.604). Hence, we calculated the full model as described in the statistical methods section, but excluded gender from the further analysis (it comprised mean fixation duration as dependent variable, objects and group as fixed effects with a cross-level interaction between both (to obtain the differences in the respective influences), and stimulus number and subject as cross-classified levels with random slopes of objects in both). The estimates for the three groups [**Table 3**, model 3 (b)] show that the mean duration of the single gaze points was also for the sticks shorter in the orangutan group than in the two human groups. Within the individual groups, almost all participants had shorter mean gaze point durations for the unmarked sticks, with the greatest differences in the two human groups (see also **Figure 8** for the illustration of the differences between the three groups and between the single participants). The results for the mean gaze points were calculated with a log-transformed dependent variable as described in the Section “Materials and Methods.”

### Aesthetic Preference Test

The aesthetic preference test revealed that for the hand axes, the aesthetic evaluation was in accordance with the fixation preference in 50.27% (Cohen’s κ = 0.94) of the cases for the German group and in 50.58% (Cohen’s κ = 0.86) of the cases for the Namibian group. In the German group, seven participants pointed more often to the marked hand axes as more beautiful and 18 participants to the unmarked hand axes. In the Namibian group, 10 participants pointed more often to the marked hand axes and 8 to the unmarked hand axes for their aesthetic evaluation. For the sticks, the aesthetic evaluation was in accordance with the fixation preference in 57.88% (Cohen’s κ = 1) of the cases for the German group and in 52.83% (Cohen’s κ = 0.87) of the cases for the Namibian group. 16 of the German participants pointed more often to the marked sticks and 9 to the unmarked sticks, while 13 in the Namibian group pointed more often to the marked sticks and 5 to the unmarked sticks.

### Spatial Analysis

In addition to the general linear mixed models for the temporal effects of the viewing behavior of the three groups (preference measured in fixation duration, number of fixations, mean duration of the gaze points), we also inspected the spatial aspects of their viewing behavior which can be interpreted from the descriptive mean values (**Table 4**). Comparing the large AOI with the small AOI, it is clear that the three groups had a different viewing pattern regarding the perception of the object in relation to its background. The orangutans had the greatest loss of fixation points and fixation duration when the AOI was placed only around the object, which indicates that they scanned the entire background and perceived the object as part of the background with the same attention (**Table 4**: total duration of fixation on stimulus compared to total duration of fixation on stimulus–small AOI).

**Table 4:** Hand axes and sticks: descriptive mean values for small and large areas of interest (AOIs).

	Germans		Namibians		Orangutans	
AOI	Large	Small	Large	Small	Large	Small
**Hand axes**
Radial distance (pixels)	75		106		143	
Fixation on stimulus						
Number	7.10	6.78	5.52	4.95	4.45	3.52
Total duration (s)	2.91	2.83	2.52	2.25	1.57	1.27
Fixation on marked stimulus						
Summed duration (s)	1.68	1.65	1.31	1.19	0.84	0.67
**Sticks**						
Radial distance (pixels)	84		109		152	
Fixation on stimulus						
Number	7.12	6.11	5.66	3.94	4.42	2.67
Total duration (s)	2.91	2.65	2.60	1.84	1.41	0.95
Fixation on marked stimulus						
Summed duration (s)	1.80	1.67	1.49	1.08	0.79	0.60

*The table shows the descriptive mean values regarding the general viewing behavior, giving the mean number of fixations on each stimulus, the mean total duration of fixation on each stimulus in seconds (s) and the mean summed fixation duration for each marked object in seconds. The summed fixation duration for unmarked objects is derivable from the difference between the values for the whole stimulus and for the marked object. The same values are then given for the small area of interest (AOI). The radial distance is calculated from the object center independent of the AOI*.

**FIGURE 9:**
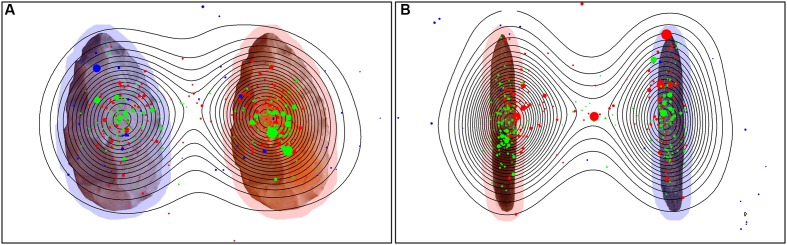
**Global heat map for hand axes (A)** and sticks **(B)**. Stimuli with the fixation points of all participants of all three groups. Green represents the German group, red the Namibian group and blue the orangutans. Contour lines indicate the density of the aggregated fixation duration of all participants. The size of the single gaze points represents their duration, with a larger size for a longer duration. The marked objects are surrounded with a red shadow and the unmarked objects with a blue shadow to improve their recognizability.

However, humans in general paid more attention to the object, with a comparison of the Namibians and the Germans showing that the Germans scanned only the object and paid no attention to the background while the Namibians analyzed the object in combination with its background. These data are illustrated in **Figure 9** (global heat maps for hand axes and sticks), which shows the aggregated fixation points of all participants, colored differently for the three groups. Blue (black) dots represent the fixation pattern of the orangutans, red (dark gray) dots that of the Namibians and green (light gray) dots that of the German participants. It is clear that the orangutans had the largest distribution over the objects and the Namibians paid more attention to the object–background relation, because the red dots are distributed beyond the objects, while the German participants concentrated on the object centers. To confirm these results, we calculated a radial distance from each object center (**Table 4**) to analyze how large the viewing patterns of the three groups were. This radius confirms our findings from the comparison of the small and large AOIs. The orangutans had the largest radius (143 pixels for hand axes, 152 for sticks), the Germans had the smallest (75 pixels for hand axes, 84 for sticks) and the Namibians were in between (106 pixels for hand axes, 109 for sticks).

## Discussion

In the present study, we investigated the patterns of fixation on marked and unmarked objects for two culturally different human groups and one species of non-human great apes, to determine whether decorated objects were preferred. We found a fixation preference for marked (decorated) objects in both human groups in relation to both the hand axes and the sticks, while the orangutans tended to prefer only the marked sticks. Among the Namibians, the preference for marked objects was less pronounced for the hand axes but very strong for the sticks. All groups fixated on marked hand axes and sticks more frequently and for longer durations than unmarked objects. This difference in the gazing pattern for marked and unmarked objects was the strongest in the German group. Orangutans had shorter single gaze point duration and larger saccades than both human groups, which supports the findings of our previous studies (Mühlenbeck et al., 2015, 2016) and of Kano et al. (2011). Together, our findings showed that humans, independent of their cultural background, processed the stimuli by using the markings as points to return to (visible in the longer fixation duration and the higher number of fixations), which supports our hypothesis of a shared human ability to let the attention be directed by features to potentially meaningful points. These findings are also consistent with the findings of one of our previous studies involving a cultural and species comparison of fixation preferences for symmetric versus asymmetric structures. We found that in contrast to orangutans, the two tested human cultures preferred symmetric patterns over asymmetric ones in their visual processing (Mühlenbeck et al., 2016). However, one cultural difference we found in the present study is that Namibians paid more attention to the object–background relation than did Germans. A reason for this could be that populations that spend most of their daily lives in the outdoors have better depth perception, which is linked to the perception of the background of an object (Cordain et al., 2002).

Orangutans differentiate objects or tools according to their cultural knowledge (Gruber et al., 2012), whereby this cultural knowledge is acquired rather through observational learning than imitative learning, i.e., orangutans rather seem to learn something about the results a tool can produce than about the behavioral strategies of the tool user (Call and Tomasello, 1994). For the group living at WKPRC, sticks are familiar and frequently used tools. We found that these apes seemed to attend only to the markings on these already known objects, as indicated by the higher fixation preference value for the sticks than for the hand axes. A possible explanation could be that for the orangutans, the emphasis did not primarily lie on the markings but on the objects themselves, and that when confronted with known objects, they attended to the markings as something different or new. Confronted with unknown objects (the hand axes), they did not attend to the markings, which indicates that the marking *per se* was not the attention-attracting feature. It seems that sticks but not rocks or markings are intrinsically salient to orangutans, and that because sticks are salient, the details (i.e., markings) become salient. For humans, in contrast, it seems that markings are intrinsically salient, and then anything marked becomes salient. The benefit that could have arisen for humans from the invention of markings lies in the possibility of storing information (for oneself and for others) in the manipulated objects, which is one prerequisite for creating external symbolic storage. If producer and observer of the marked object are aware of their joint attention, it seems obvious to use this attention permanently for giving this marking an abstract meaning. In this way, markings could have been an opportunity, in addition to gestures and vocalizations, to direct others’ attention. As Donald pointed out regarding the invention of external symbolic storage, “a technological bridge was under construction that would eventually connect the biological individual with external memory architecture,” (Donald, 1991, p. 284). Donald described this technological bridge in regard to pictorial images, but it should also be seen in the first marking behavior, as the precursor of pictorial images. The invention of markings could have made it possible to direct the attention of others, store information, and transfer information to others, which also strengthens social bonds when combined with ritual behavior (Boyer and Liénard, 2006) and which in turn may have contributed to the demographic expansion of *Homo sapiens* (Mellars, 2006; Cela-Conde et al., 2009).

We assumed that this structuring behavior would match a parallel aesthetic appreciation, as measured by our aesthetic preference test, but we did not clearly find such a match. Reasons for this could be that the subjects have personal preferences for specific structures, or that aesthetic preferences could be influenced by family- or in-group relations (like attentional preferences – for a discussion on how social saliency drives visual orienting behavior, please see Shepherd and Platt, 2009). In our statistical models, these personal preferences are mirrored in the random effects, but personal preferences may also have affected the aesthetic evaluations. We selected hand axes and sticks with different forms and decorated them with different markings and incisions to cover a wide range of possible structures. It is possible that the participants used the markings for their practical benefit, as the markings helped to structure the visual field by making the objects prominent, and that the structures that were created through the addition of the decorations on the objects did not conform to how they personally would have structured and marked the objects. In general, it is still possible that aesthetic appreciation comes along with structuring behavior, but that the objects we presented were not marked in a way that all of the viewers would regard as beautiful. It is also possible that not only our choice of the decoration and its positioning but also our choice of the stimuli was related to the low aesthetic preference for the marked hand axes and, in the case of the Namibian group, for the low fixation preference. This appears to be supported by the strong effect we found for the sticks. A reason for the strong effect of decoration on the sticks and the weaker effect for the hand axes might be that the existing structure of the unmarked hand axes was already pleasant and the marking disturbed this structure. The same would not hold for the sticks because the long form of the sticks was not so easily disrupted.

To summarize, across the two cultures, markings made objects more salient to humans (relative to unmarked matched objects and backgrounds). Humans treated markings as points to return to, which confirmed our hypothesis that marked objects direct human attention. The difference between the two cultural groups lay in the different attention they paid to the relation between the objects and their backgrounds. The species differed, insofar as the orangutans either did not use these given marked points or tended to use them only when they were already familiar with the object. Despite their longer fixation on the marked sticks, however, the orangutans’ general perception of the objects and their backgrounds was still very wide-ranged, with quick scanning. Future eye-tracking studies should examine whether the human structuring ability to treat markings as relevant for attention exists in other non-human great apes, in order to determine whether this behavior is unique to humans or is shared by other great apes. It also might be interesting to test non-human great apes with objects they had seen before with and without markings. Also, in addition to the gazing preferences, future studies should determine which species actively mark objects or order their surroundings, as well as whether and when humans regard self-structured and marked objects as beautiful and whether their aesthetic appreciation could be influenced by ownership or in-group and family relations.

## Conclusion

We aimed to investigate the origins of hominin object-marking behavior by comparing the influence of such markings on ape versus human attentional preferences and on the aesthetic preferences of humans from different cultures. We found that orangutans seem to perceive objects more in the overall context of their environment, in comparison to humans, who concentrate more on the objects themselves. Unlike the apes, humans concentrate on specific points and use markings and ordered structures for visually processing their surroundings. All of the human participants in this study were similar in their visual exploratory behavior. Given that the marked and unmarked objects were differently judged in terms of being beautiful, it seems that the way in which decorations are made leads to different aesthetic evaluations. A reason for the evolution of decorating behavior could be that the markings facilitate an individual’s structuring of his or her environment. The significance of our findings is represented in the human ability to let one’s attention be directed to the markings, which was shared in the two very distinct cultures we tested. This attention-directing effect of markings, which was found only in the two human cultures and not in the orangutans, should be considered for the further exploration of the first non-utilitarian object manipulation and the invention of external symbolic storage.

## Author Contributions

Conceived and designed the experiment: CM, TJ, and KL; Performed the experiments: CM, CP, and KL; Analyzed the data: CM; Wrote the paper: CM, TJ, and KL.

## Conflict of Interest Statement

The authors declare that the research was conducted in the absence of any commercial or financial relationships that could be construed as a potential conflict of interest.
